# Proactive Personality and Academic Engagement: The Mediating Effects of Teacher-Student Relationships and Academic Self-Efficacy

**DOI:** 10.3389/fpsyg.2021.652994

**Published:** 2021-06-08

**Authors:** Peiyao Chen, Chenye Bao, Qiyang Gao

**Affiliations:** ^1^School of Teacher Education, Shaoxing University, Shaoxing, China; ^2^Center for Brain, Mind and Education, Shaoxing University, Shaoxing, China

**Keywords:** proactive personality, academic engagement, teacher-student relationships, academic self-efficacy, elementary school students

## Abstract

A proactive personality provides students with strong competitiveness in academic learning. However, previous research primarily focused on the effects of the big five facets, and less attention was paid to proactive personality which shows more incremental validity in learning. The current study aimed to investigate the relationship between proactive personality and academic engagement. The sample consisted of 519 students (245 females, 274 males; *M*_age_ = 10.20, *SD* = 0.891). The study used Mplus 7.0 software to establish structural equation models (SEM). The results showed a significant positive relationship between proactive personality and academic engagement. Teacher-student relationships and academic self-efficacy were found to fully mediate separately between proactive personality and academic engagement. Moreover, the serial mediator model indicated that proactive personality was sequentially related to academic engagement through teacher-student relationships and academic self-efficacy. The implications for learning and teaching are discussed.

## Introduction

Academic engagement, defined as students' active participation in and emotional commitment to learning (Casuso-Holgado et al., 2013), has a critical role in academic success (Wang and Eccles, 2012; Kwon et al., 2018). That is, students with a high level of academic engagement are more likely to concentrate on learning and achieve higher academic performance (Wang and Holcombe, 2010; Kwon et al., 2018). On the contrary, those with a low level of engagement may fail exams, drop out of school, and have problems in behaviors (Fredricks et al., 2004; Chipchase et al., 2017).

Previous research found the big five personalities can significantly predict academic engagement (Bakker et al., 2015; Sulea et al., 2015; Closson and Boutilier, 2017). More specifically, openness to experience (Bakker et al., 2015) and conscientiousness (Sulea et al., 2015) were positively associated with academic engagement, while neuroticism was not significantly related to academic engagement (Closson and Boutilier, 2017). However, theorists have argued that, when trying to associate personality traits to a specific criterion, the criterion-related validity of basic personality traits maybe not be specifically suited to explaining the outcome (Hough and Schneider, 1996).

Proactive personality, which refers to a “stable disposition to take personal initiative in a broad range of activities and situations” (Seibert et al., 2001, p. 847), was found to have more incremental validity regarding motivation to learn than the big five personality traits (Major et al., 2006). Motivation to learn (i.e., the desire to engage in development activities) and academic engagement are distinct constructs, but at their essence, they share fundamental properties. As such, we speculated that proactive personality may be related to academic engagement. In the current study, we focused on examining whether students with a higher level of proactive personality tend to engage more in learning activities. In addition, certain theorists pointed out that personality might have an effect on academic behavior not directly but through behavior-related variables (Chen and Astor, 2011; Charalampous and Kokkinos, 2014). Based on the model of reciprocal causation (MRC; Bandura, 1989) and social cognitive theory (Bandura, 2007), this study also investigated the extent to which teacher-student relationships and self-efficacy act as mediators in the relationship between proactive personality and academic engagement.

## Literature Review

### Proactive Personality and Academic Engagement

Proactive personality can significantly predict academic engagement (Major et al., 2012). Students with proactive personalities are more likely to succeed than passive students (McNall and Michel, 2011). Proactive individuals tend to scan for opportunities, look for all possibilities to utilize resources, and shape the environment (Parker and Collins, 2010). They have a higher level of persistence, demonstrate initiate to overcome difficulties from unexpected environments, and involve themselves to fulfill their ambitions (Hu et al., 2020). For example, Major et al. (2012) found that students with a higher level of proactive personality were less likely to disengage and used fewer avoidant strategies to reduce effort or give up in stressful situations. Moreover, Zhu et al. (2017) found that proactive students reported higher academic performance in a stressful situation than passive students. In addition, Islam et al. (2018) found students who had a proactive personality undertook more responsibility in academic citizenship behavior and helped other students engage in extracurricular activities.

### Teacher-Student Relationships as a Mediator

A stable tendency to observe and understand the world may have an effect on how students perceive teacher behavior (Charalampous and Kokkinos, 2014). Previous research has shown that different personalities traits foster different teacher-student relationships (Zee et al., 2013; Charalampous and Kokkinos, 2014). For example, Charalampous and Kokkinos (2014) found that students with high levels of extraversion experience more teacher proximity, while those with high levels of neuroticism experience less. Furthermore, their research also found that perceiving teacher proximity had an effect on students' achievement and simultaneously mediated the relationship between personality traits and students' achievement. According to MRC, “behavior, cognition, and other personal factors, and environmental influences, all operate as interacting determinants that influence each other bidirectionally” (Bandura, 1989, p. 2). Personal factors may influence individuals' behavior, as well as how they perceive the environment. Teacher-student relationships, as a crucial role in helping students to solve problems, may be a potential candidate.

Teacher-student relationships refer to students' sense that they have positive interactions with their teachers, and that their teachers are supportive of their learning needs (Collie et al., 2016). A large number of studies found that teacher-student relationships were associated with academic engagement (Gehlbach et al., 2012; Pianta et al., 2012; Varga, 2017; Hughes and Cao, 2018). As students spend at least one-quarter of their waking time in school, teacher-student relationships provide the potential for classroom resources and catalyze important benefits for student engagement in class (Hughes et al., 2008). Numerical research has also well documented the relationship between teacher-student relationships and academic engagement across different educational contexts and ethnicities (Den Brok et al., 2010; Christenson et al., 2012; Pianta et al., 2012). Moreover, Chen and Astor (2011) recruited more than 14,000 students from Taiwan and found that students with negative personalities were less likely to build teacher-student relationships, which further inhibited students engaging in learning.

Proactive personality may be a key internal factor in dealing with teacher-student relationships. Theoretically, students with proactive personalities may actively ask teachers questions, share life experiences with teachers, obey classroom rules, and comply with class discipline to build good teacher-student relationships. However, to the best of our knowledge, no study explores the relationship between proactive personality and teacher-student relationships. There were several indirect evidences (Sanchez-Cardona et al., 2012; Charalampous and Kokkinos, 2014; Hua et al., 2020; Hu et al., 2020). For example, Hu et al. (2020) found that students with a proactive personality tend to show more psychological comfort, acquaintance their with social environment (i.e., like interacting with professors), and social adjustment.

### Academic Self-Efficacy as a Mediator

According to the social cognitive theory that individuals should be concerned “not with what one has, but with the belief in what one can do with whatever resources one can muster” to carry out tasks (Bandura, 2007), academic self-efficacy may be another mediator between proactive personality and academic engagement. Academic self-efficacy is defined as students' judgment of their own capabilities to carry out school-related activities (Schunk, 1991), which equips students for powerful thinking and confidence (Bandura, 1986). Existing research revealed that academic self-efficacy was positively related to academic engagement (Bandura, 1977; Sanchez-Cardona et al., 2012; Siu et al., 2014; Martin and Rimm-Kaufman, 2015; Olivier et al., 2019; Ozkal, 2019). Students with high academic self-efficacy are more willing to spend extra energy and time to complete learning tasks; thus, they concentrate more on school-related activities (Siu et al., 2014). Contrastingly, students with low self-efficacy tend to dwell on past mistakes and reduce their efforts when facing difficult tasks (Bandura, 1977). For example, Ozkal (2019) found that self-efficacy for learning could significantly predict academic engagement. Moreover, one study with 387 fifth-grade elementary school students indicated that if a student enters the classroom environment with strong internal resources (e.g., self-efficacy), the student may be well-equipped to face the challenges presented, resulting in more learning engagement (Martin and Rimm-Kaufman, 2015). Additionally, Sanchez-Cardona et al. (2012) conducted longitudinal research and reported that from 37 students, those with higher openness traits tended to consider demands as challenges and used all available resources to promote engagement.

Proactive personality can significantly predict academic self-efficacy (Lin et al., 2014; Hua et al., 2020). Students who have a higher level of proactive personality are more likely to build a strong belief that they can succeed, which contributes toward them achieving goals (Lin et al., 2014). For example, Lin et al. (2014) conducted a longitudinal study and found that students possessing highly proactive personalities were more likely to utilize available resources to improve situations and persistently achieve goals. Furthermore, they found that a proactive personality could predict later academic self-efficacy. Moreover, Hua et al. (2020) found that students with a proactive personality showed a higher level of adjustment self-efficacy, which further influenced their academic behavior.

### Sequential Pathway

Proactive personality may influence academic engagement through teacher-student relationships and academic self-efficacy sequentially. Theoretically, proactive students tend to create an optimal environment and build better relationships with teachers. Supportive teacher-student relationships provide care, attention, and positive emotion for students to develop a healthy learning psychology and build self-confidence, establishing a higher self-efficacy (Xu and Qi, 2019). With a fruitful environment and powerful motivation, students are more willing to engage in school-related activities. Therefore, the current study hypothesized that personality variables may influence behavior through environment and internal variables.

### The Current Study

In China, elementary school students frequently face fierce competition and high pressure to pass exams, obtain a good public ranking of academic performance, and complete a high amount of homework (Hesketh et al., 2010). Under these circumstances, better engagement helps them to reach an outstanding performance and obtain academic success (Caruth, 2018). Thus, understanding the ways in which we can engage elementary school students in their education is important.

**Figure 1:**
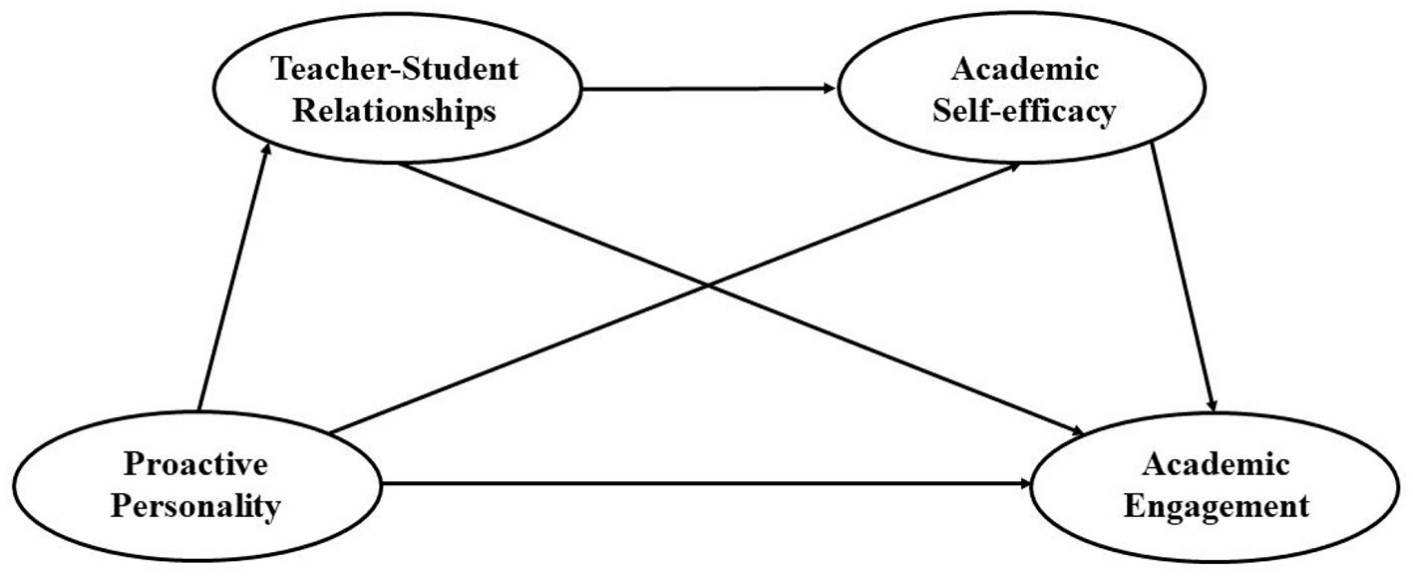
The proposed mediation model.

On the basis of the MRC, social cognitive theory, and previous research, the current study hypothesized that proactive personality would predict academic engagement (H_1_), and the relationship would be mediated by teacher-student relationships (H_2_) and academic self-efficacy (H_3_). Moreover, teacher-student relationships and academic self-efficacy could mediate this link sequentially (H_4_). Structural model as follows (Figure 1).

## Methods

### Participants and Procedure

In this study, 519 participants were recruited from two elementary schools in Yue Cheng District, Shaoxing City, China. Mean participant age was 10.29 years (*SD* = 0.849) from grade four to five, with a range of 9 to 14 years; 274 (52.8%) were boys and 245 (47.2%) were girls.

The present study obtained students and school consent as well as ethical approval from the ethical committee of Shaoxing University. The reference code corresponding to the approval of the research by the ethics committee was 2021-002. Considering that children's vocabulary and reading skills has an impact on their understanding of questionnaire items, the study recruited students from the fourth and fifth grades, who master 2500 to 3000 words and comprehend the meaning and emotional expression of sentences (Xiong, 2004, p. 77). In addition, as the Modern Chinese Word List showed that 2500 words cover 97.97% of written documents (Lou and Wang, 1987, p. 3), students from grade four to five may be unable to complete self-report scales. The questionnaire was administrated in a classroom setting. Students were first required to provide their demographic information. Thereafter, the research assistant instructed them to complete scales that assessed proactive personality, teacher-student relationships, academic self-efficacy, and academic engagement. The time taken to complete each scale was 25, 30, 30, and 25 min, respectively.

### Measures

#### Proactive Personality Scale

The current study recruited professional translators to translate the Proactive Personality Scale, developed by Bateman and Crant (1993). The scale has 17 items (e.g., “I am always looking for new ways to improve the quality of my life”). Respondents indicated the extent to which they agreed with each statement on a 7-point Likert-type scale ranging from 1 (*strongly disagree*) to 7 (*strongly agree*). A high score indicates a more proactive personality. This Chinese version was also used by Gao et al. (2019) with Chinese elementary school students and had good fit (χ^2^/*df* = 2.785, *CFI* = 0.907, *TLI* = 0.893, *RMSEA* (90% CI) = 0.054, *SRMR* = 0.05). Cronbach's α was 0.89 in the present study.

#### Teacher-Student Relationships Scale

The Teacher-Student Relationships Scale (TSRS) was originally proposed by Pianta (1999). The TSRS consists of 23 items assessing the following four factors: closeness (e.g., “I have a close and warm relationship with my teacher”), conflict (e.g., “The teacher always seems to have a conflict with me”), dependency (e.g., “The teacher is willing to explain to me when I meet difficulties in my study”), and satisfaction (e.g., “I am very satisfied with my relationship with my teacher”). The scores of conflict need to be reversed. Participants rated items in terms of how applicable each statement was to their relationship with their current teachers, using a 5-point Likert-type scale ranging from 1 (*definitely does not apply*) to 5 (*definitely applies*). This questionnaire had good fit (χ^2^/*df* = 2.840, *CFI* = 0.920, *TLI* = 0.910, *RMSEA* = 0.060, *SRMR* = 0.062) and cronbach's α was.81,0.86,0.81,0.66 for closeness, conflict, dependency, and satisfaction sequentially in the present study.

#### Academic Self-Efficacy Scale

The Academic Self-Efficacy Scale was originally developed by Pintrich and De Groot (1990) and revised by Liang (2000). This scale consists of 22 items and two subscales: learning ability efficacy (e.g., “I believe I can get good grades when I study”) and learning behavior efficacy (e.g., “I often find that when I am reading a book, I don't know what it means”). Item responses ranged from 1 (*definitely disagree*) to 5 (*definitely agree*). The academic self-efficacy scale had good fit (χ^2^*/df* = 3.510, *CFI* = 0.952, *TLI* = 0.943, *RMSEA* = 0.070, *SRMR* = 0.043). The learning ability efficacy (α = 0.90) and learning behavior efficacy (α = 0.70) had satisfactory reliability.

#### Student Engagement Scale

The Student Engagement Scale was developed by Lam et al. (2009). The scale consists of 16 items, and we adopted the following three subscales: cognitive engagement (e.g., “When I study, I construct new knowledge with my own experience”), behavioral engagement (e.g., “I work hard at school”), and emotional engagement (e.g., “I was in high spirits while studying in class”). Answers are rated on a 5-point Likert scale ranging from 5 (*definitely agree*) to 1 (*definitely disagree*). The academic self-efficacy scale had good fit (χ^2^*/df* = 3.460, *CFI* = 0.904, *TLI* = 0.891, *RMSEA* = 0.069, *SRMR* = 0.056). Cronbach's α was.79,0.90, and 0.90 for cognitive engagement, behavioral engagement, and emotional engagement, respectively.

### Data Analysis

The software SPSS 25.0 was used to conduct some basic analyses, including descriptive statistics and correlations for proactive personality, teacher-student relationships, academic self-efficacy, and academic engagement. All variables were computed and descriptive statistics, namely mean (M) and standard deviation (SD), for each variable and correlations between variables were obtained. Mplus 7.0 software (Muthén and Muthén, 2012) was used to establish structural equation models (SEM). In the current study, a bootstrapping analysis was conducted with proactive personality as the independent variable, academic engagement as the outcome variable, teacher-student relationships and academic self-efficacy as mediators, and gender and age as covariates),with 5000 resamples to test a serial mediation model and to calculate the 95% CIs. The numbers of subdimensions in each scale was unequal; thus, mean scores of the items were used for all observable variables in this study.

## Results

### Common Method Bias

In order to control the common method bias, the present study took measurements, including instruction, an anonymous survey, and random order of items. After collecting data, this study used Harman's single-factor analysis. Results found that the total variance extracted by one factor was 25.349%, which was less than the recommended threshold of 50%.

### Descriptive Statistics

Table 1 presents descriptive statistics and correlations analyses between control variables, proactive personality, teacher-student relationships, academic self-efficacy, and academic engagement. No data were missing. The mean scores of proactive personality, positive teacher-student relationships, negative teacher-student relationships, academic self-efficacy, and academic engagement ranged from 2.05 to 5.01. Except for gender and grade, correlations between all main variables were significant and positive.

**Table 1:** Descriptive statistics and correlations among variables.

	***M* ±*SD***	**1**	**2**	**3**	**4**	**5**
1. PP	5.01 ± 0.91	1				
2. PTSRs	3.54 ± 0.75	0.433^***^	1			
3. NTSRs	2.05 ± 0.86	−0.104^*^	−0.382^***^	1		
4. ASE	3.62 ± 0.57	0.489^***^	0.425^***^	−0.117^**^	1	
5. AE	4.09 ± 0.73	0.381^***^	0.438^***^	−0.214^***^	0.542^***^	1

*PP, proactive personality; PTSRs, positive teacher-student relationships; NTSRs, negative teacher-student relationships; ASE, academic self-efficacy; AE, academic engagement*.

* *p < 0.05;*

** *p < 0.01;*

*** *p < 0.001*.

### Structural Equation Model

First, prior to the analysis of the indirect effects model, we used a total direct model to derive the effects of proactive personality on academic engagement. Following our correlation results, we controlled for gender and grade by connecting them to the endogenous variables (proactive personality and academic engagement). The model revealed a good fit to the data: χ^2^*/df* = 2.81; comparative fit index (CFI) = 0.988; Tucker Lewis index (TLI) = 0.976; root mean square error of approximation (RMSEA) = 0.059; standardized root means square residual (SRMR) = 0.017. Gender and grade were the control variables in the direct model. Gender reported a positive effect on academic engagement (β = 0.137, *p* < 0.01), whereas grade showed a negative effect on academic engagement (β = −0.162, *p* < 0.001). The results revealed that proactive personality could significantly and directly promote academic engagement (β = 0.40, *p* < 0.001).

### Multiple Indirect Effects Model

**Figure 2:**
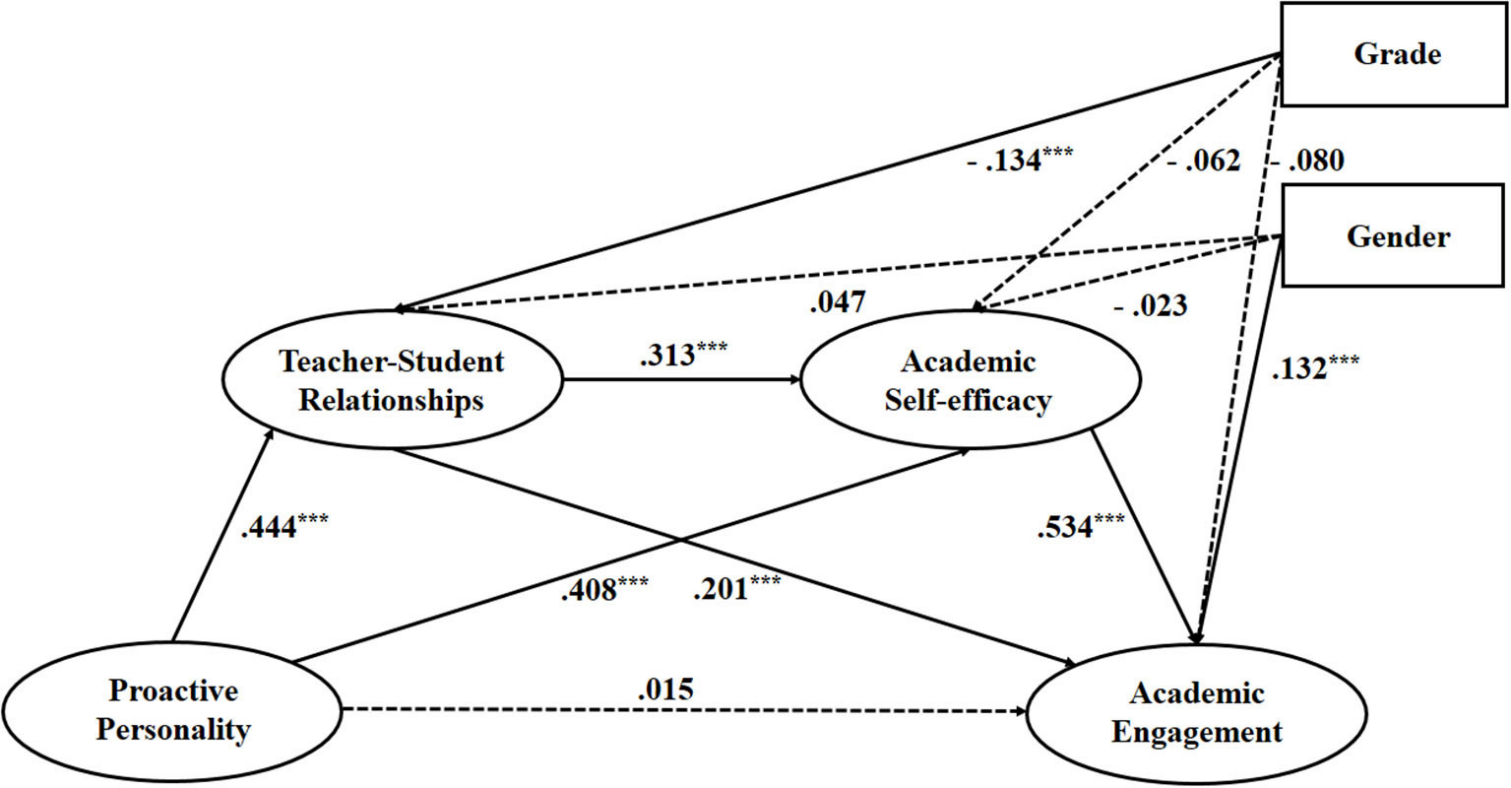
The serial mediation model with teacher-student relationship and academic self-efficacy as mediators of the linkage between proactive personality and academic engagement.

Second, we established the multiple indirect effects model and examined the effects of proactive personality on academic engagement through mediators, namely academic self-efficacy and teacher-student relationships. Further, we also controlled for gender and grade in this model. The multiple mediation models also demonstrated an acceptable fit: χ^2^/*df* = 4.02, *CFI* = 0.952, *TLI* = 0.928, *RMSEA* = 0.076, *SRMR* = 0.035. Figure 2 displays the influential paths in detail. Table 2 displays direct and indirect effects and their associated 95% confidence interval (CI). For the control variables, gender had a significant positive effect on academic engagement (β = 0.132, *p* < 0.001) and grade had a non-significant effect on academic engagement (β = −0.080, *p* = 0.055). The size of the direct effect of proactive personality on academic engagement was not significant, indicating that there was full mediation between proactive personality and academic engagement in the multiple indirect effects model. All other links in this model were significant (β ranging from 0.20 to 0.53, *p* < 0.01) (Figure 2).

**Table 2:** Bootstrap analyses of magnitude and statistical significance of indirect effects.

**Model pathways**	**Effect**	***p***	**95%*****CI***	
			**Lower**	**Upper**
PP → TSRs → AE	0.089^***^	<0.001	0.141	0.322
PP → ASE → AE	0.218^***^	<0.001	0.043	0.123
PP → TSRs → ASE → AE	0.074^***^	<0.001	0.033	0.081
PP → TSRs/ASE → AE	0.385^***^	<0.001	0.033	0.153
(total indirect effect)				
PP → AE (direct effect)	0.015	=0.056	−0.097	0.122
PP → AE (total effect)	0.397^***^	<0.001	0.314	0.475

PP, proactive personality; TSRs, teacher-student relationships; ASE, academic self-efficacy; AE, academic engagement. *p < 0.05; **p < 0.01;

*** *p < 0.001*.

As shown in Table 2, the total mediating effect of proactive personality on academic engagement was 0.385. The size of the mediating effect through academic self-efficacy was larger than that through teacher-student relationships, at 0.218 and 0.089, respectively. They accounted for 54.91 and 22.42% of the total indirect effects. It indicated that the association between proactive personality and academic engagement was largely mediated by teacher-student relationships. However, the size of the mediating effect through positive teacher-student relationships and academic self-efficacy was relatively small, and only accounted for 18.64% of the total effects. Above all, the mediating effect made up 96.98% of the total effect, indicating that proactive personality can significantly and positively affect academic engagement through these two mediators (teacher-student relationships and academic self-efficacy). It implied that teacher-student relationships and academic self-efficacy were essential mediators between the proactive personality and students' academic engagement, especially teacher-student relationships.

## Discussion

The aim of the present study was to examine the relationship between proactive personality and academic engagement through teacher-student relationships and academic self-efficacy among Chinese elementary school students. Structural modeling results demonstrated that proactive personality directly predicted academic engagement. In addition, teacher-student relationships and academic self-efficacy mediated the effect of proactive personality on academic engagement. Finally, the serial two-mediator model indicated that proactive personality influenced academic engagement via teacher-student relationships and academic self-efficacy sequentially.

The findings showed that proactive personality was significantly related to academic engagement, which supported H_1_. This finding is consistent with Major et al. (2012), confirming that students with a high level of proactive personality tend to engage more in studying. Furthermore, unlike previous studies focusing on undergraduates (Major et al., 2012; Islam et al., 2018), the current study extends the scope of the sample to elementary school students, which suggests the importance of cultivating these personality traits in children from an early age. In addition, previous research primarily paid attention to the effect of the big five model (Bakker et al., 2015; Sulea et al., 2015; Closson and Boutilier, 2017); our study extends the personality traits and firstly explores the relationship between proactive personality and academic engagement.

The results also showed that teacher-student relationships fully mediated the relationship between proactive personality and academic engagement. That is, students with a proactive personality were more likely to have positive teacher-student relationships. When students perceived positive relationships with their teachers, they had higher levels of academic engagement, which supported H_2_. To some degree, this finding also supported MRC model, which was in line with the application of MRC model by Charalampous and Kokkinos (2014). Compared to the previous research (Charalampous and Kokkinos, 2014), the present study chooses another personality trait and also gets the same model. The most obvious trait of proactive personality involves changing the environment (Bateman and Crant, 1993; Hu et al., 2020). Students with proactive personalities are more willing and open to interact with teachers and establish positive teacher-student relationships, which contributes to engaging and completing tasks.

Additionally, the study also showed that academic self-efficacy fully mediated the relationship between proactive personality and academic engagement. More precisely, students with proactive personalities experience more academic self-efficacy (Lin et al., 2014), which leads them to become more confident to face challenges and engage in their school-related activities (Gehlbach et al., 2012). The current study further explored the findings of Lin et al. (2014) and first found the mediating path in a school setting. For students, academic self-efficacy is an essential mediator during the learning process (Hua et al., 2020). This result indicates that a higher proactive personality triggered higher motivation to engage in academic activities.

Finally, our findings revealed the relationship between proactive personality and academic engagement through teacher-student relationships and academic self-efficacy as mediators in sequence, which is consistent with H_4_. To be specific, proactive students who had positive teacher-student relationships experienced higher academic self-efficacy, which increased their academic engagement. Students with a proactive personality obtained resources and established supportive teacher-student relationships. When students perceived support from their teachers, they experienced a sense of security and developed high self-efficacy (Xu and Qi, 2019). This can also lead them to feel that they were part of the classroom and school community (Martin and Rimm-Kaufman, 2015).

Some limitations of the present study should be considered. First, this study had a cross-sectional design, which does not allow us to make causal inferences. Thus, future research is needed to replicate the present findings using longitudinal or experimental designs to determine causal relationships between the research variables. Second, teacher-student relationships were collected from students' perspective. Future research can consider teachers' perspective or teacher-student congruence. Third, we chose teacher-student relationships as a mediator; however, other environmental effects, such as that of peers, need to be taken into consideration. Finally, as participants were not required to report on the relationship with specific teachers, future studies could focus on specific school subjects and explore whether there are differences between them.

There are several practical implications of the results of the present study. Proactive personality plays an important role in Chinese students' academic performance (Zhu et al., 2017). Given that, elementary schools and their teachers should value the cultivation of students' proactive personality. At first, teachers should take measures to help develop the proactive characteristics of students, whose personality development is not yet complete (Zhu et al., 2017). For example, in class, teachers could encourage less proactive personality students to answer questions or show their ideas. After class, teachers could encourage less proactive students to attend different activities and interact with instructors more often. Furthermore, empowerment programs, which are positively related to proactive personality, should be allowed (Judge and Ilies, 2002). For example, schools could invite counselors to train students in “the art of strategic thinking”, which could significantly improve students proactive thinking and proactivity (Kirby et al., 2002).

The second implication of the present study is that positive teacher-student relationships could positively influence academic engagement. According to this, maintaining strong teacher-student relationships is important, which requires teachers and students to mutually interact (Yücel et al., 2010). From the teachers' perspective, teachers should consider students' perspectives and negotiate with them, instead of holding absolute authority, to achieve educational goals and protect the proactive personality of students (Alderman and Green, 2011). From the students' perspective, students could share their personal experiences, such as interesting family stories, with teachers to strengthen the relationship (Baker et al., 2008).

Our findings also indicate that enhancing students' academic self-efficacy could be an effective strategy to promote students' engagement. Teachers could design simple questions to increase students' probability of success in class and increase their self-efficacy (Walker, 2003). In addition, teachers need to encourage students and reduce overwhelming competition among students to avoid failure and protect their academic self-efficacy. Students should also attribute their failure to changeable and controllable factors, such as effort, to protect their self-efficacy.

## Conclusion

Despite the above limitations, the current study has theoretical and practical implications. First, it complements existing research on the effect of personality traits on academic engagement. Second, we applied the MRC and examined new pathways, revealing the mediating effect of teacher-student relationships, and used social cognitive theory to introduce academic self-efficacy in the association between proactive personality and academic engagement. Third, from a practical perspective, the positive relationship between proactive personality and academic engagement indicates that parents and teachers should cultivate children's proactive personality from an early age. Additionally, as teacher-student relationships and academic self-efficacy mediate the relationship between proactive personality and academic engagement, educators should establish sufficiently positive teacher-student relationships and protect students' academic self-efficacy so that proactive individuals can actively engage in learning activities.

## Data Availability Statement

The raw data supporting the conclusions of this article will be made available by the authors, without undue reservation.

## Author Contributions

All authors listed have made a substantial, direct and intellectual contribution to the work, and approved it for publication.

## Conflict of Interest

The authors declare that the research was conducted in the absence of any commercial or financial relationships that could be construed as a potential conflict of interest.
